# A203 EARLY PROACTIVE DRUG MONITORING STRATEGY OF INFLIXIMAB AS MONOTHERAPY FOR INFLAMMATORY BOWEL DISEASE IN PAEDIATRIC PATIENTS IS ASSOCIATED WITH GOOD SUSTAINED CLINICAL REMISSION

**DOI:** 10.1093/jcag/gwac036.203

**Published:** 2023-03-07

**Authors:** C Girard, S Ackhar, S Sassine, L Chapuy, P Jantchou, C Deslandres

**Affiliations:** 1 Paediatric Gastroenterology , CHU Sainte-Justine; 2 Faculté de Médecine , Université de Montréal; 3 Paediatric Gastroenterology , Montreal Children's Hospital, Montreal , Canada

## Abstract

**Background:**

**Monotherapy with Infliximab (IFX) can be as efficient as combotherapy** with immunomodulators in the treatment and maintenance of remission for children with inflammatory bowel disease (IBD) if an **early proactive therapeutic drug monitoring strategy** is adequately performed. This strategy may allow optimization of blood levels of IFX in order to obtain a sustained clinical response.

**Purpose:**

This study demonstrated that with appropriate early trough levels of IFX before dose #3 and dose #4 , monotherapy was very efficient in inducing remssion at week 52 .

**Method:**

**A retrospective study was conducted at CHU Sainte-Justine, Montréal,Canada .Children with IBD 2 to 18 years old diagnosed between 01/2018 and 06/2020 and treated with IFX less than 30 days after diagnosis were included .IFX blood levels were collected before the 3rd and/or 4th dose of IFX and regularly thereafter. Adjustments were done in IFX dose per infusion according to blood levels and clinical response. The primary outcome was clinical remission at one year after diagnosis.The secondary outcomes included: (1) At 52 weeks, the median (IQR) dose of IFX (mg/kg) and the intervals between IFX infusions ; (2) the median (IQR) number of IFX dose changes and the median (IQR) number of blood trough levels of IFX done.**

**Result(s):**

101 patients were included : 56.4% males; 81CD; 18 UC; 2 IBDU. Mean age at diagnosis was 13.2 years (IQR = 11.20 to 15. 20).

Median time to IFX initiation after diagnosis was **5 days** (IQR :3-14).Median IFX dose #1: was 8.4 mg/kg (IQR = 5. 8 to 10).

90% of patient had an IFX optimisation (increasing dose and/or shortening intervals) after dose 3 or dose 4. **At week 52, 36,5% of patients were receiving IFX infusion every 4 weeks and 30,6% every 6 weeks**. The **median IFX dose per infusion** was **8,9mg/kg (IQR = 7.4- 9,8 ).** The IFX doses at week 52 varied greatly according to age at diagnosis The median number of IFX blood level dosage was 4 per patient over a year (IQR=3-5).**At week 52, 83 patients (84.6%) achieved clinical remission with a median IFX level of 10.96 (7.05-15.59). 74 /83 (89%) were on monotherapy and 9/83(10.8%) on combotherapy**

**Image:**

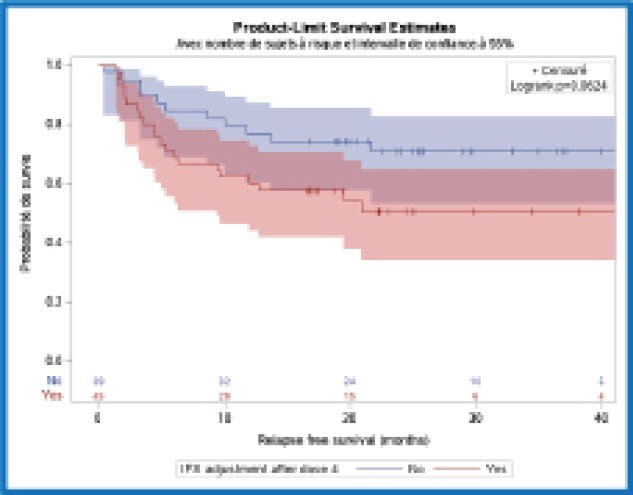

**Conclusion(s):**

**Early treatment for IBD with IFX as monotherapy and an early proactive optimization strategy is associated with a good sustained steroid free clinical remissionAt week 52, 83 patients (84.6%) achieved clinical remission with a median IFX level of 10.96 (7.05-15.59). 74 /83 (89%) were on monotherapy and 9/83(10.8%) on combotherapy.The majority of the patients required IFX optimization during their first year of treatment. We therefore recommend to proactively monitor blood levels of IFX before the third and fourth dose of IFX and thereafter, in order to lower the risk of treatment failure and anti-infliximab antibodies occurrence.**

**Please acknowledge all funding agencies by checking the applicable boxes below:**

None

**Disclosure of Interest:**

C. Girard: None Declared, S. Ackhar: None Declared, S. Sassine: None Declared, L. Chapuy: None Declared, P. Jantchou: None Declared, C. Deslandres Speakers bureau of: moderator and speaker Abbvie and Janssen

